# Low Grade Appendiceal Mucinous Neoplasm with Pseudomyxoma Peritonei: An Enigma for Pathologist

**DOI:** 10.5005/jp-journals-10018-1115

**Published:** 2014-07-28

**Authors:** Shagufta Qadri, Kiran Alam, Feroz Alam, Veena Maheshwari

**Affiliations:** 1 Department of Pathology, Jawaharlal Nehru Medical College, Aligarh, Uttar Pradesh, India

**Keywords:** Appendix, Mucinous neoplasm, Pseudomyxoma peritonei, Adenocarcinoma.

## Abstract

Malignant mucinous neoplasms of the appendix is an infrequently encountered entity. Extra-appendiceal spread of these tumor is one of the commonest etiology of pseudomyxoma peritonei, which demands a hightened vigilance in their early diagnosis. Although low-grade appendiceal mucinous neoplasms (LAMNs) largely stay confined to the appendix, but they can spread to the peritoneum as pseudomyxoma peritonei leading to an unpredictable outcome. Due to the rare occurrence of low-grade appendiceal neoplasm only tenuous and limited information is present in the medical literature. We report a case of LAMN with pseudomyxoma peritonei in a 45-year-old male, who presented with the complaints of abdominal distension associated with abdominal pain and constipation. Clinical examinations and computed tomography (CT) scan were suggestive of pseudomyxoma peritonei. Peroperative findings and histopathological examination rendered a conclusive diagnosis of low-grade appendiceal neoplasm.

**How to cite this article:** Qadri S, Alam K, Alam F, Maheshwari V. Low Grade Appendiceal Muci-nous Neoplasm with Pseudomyxoma Peritonei: An Enigma for Pathologist. Euroasian J Hepato-Gastroenterol 2014;4(2):113-116.

## INTRODUCTION

Low-grade appendiceal mucinous neoplasms (LAMNs) are rare condition with the reported prevalence of less than 1% of all appendectomies.^1^ Low-grade mucin-producing tumors of the appendix that includes adenomas and mucinous tumors of unknown malignant potential (MTUMP) may present with pseudomyxoma peritonei due to their potential to spread to the peritoneal cavity and viscera in the form of mucinous deposits.^2^ Peritoneal mucinous carcinomas with intermediate and incongr uent features are observed frequently^3^ and therefore an alternate divaricate classification has been recommended, classifying the pseudomyxoma peritonei of appendiceal origin into mucinous carcinoma peritonei—low-grade and mucinous carcinoma peritonei—high grade.^4^

Low-grade mucinous appendiceal neoplasms are considered as an enigmatic tumor because of their unpredictable clinical outcome that poses a formidable diagnostic challenge. We hereby report this uncommon case of LAMN as it bespeak the potential of this tumor to spread to the peritoneal cavity causing pseudomyxoma peritonei that would lead to a disastrous clinical outcome.

## CASE REPORT

A 45-year-old male patient not known to have any previous medical illnesses, reported to the surgery outpatient department with a clinical history of severe pain in right lower abdomen, with abdominal distension and constipation since 2 days. The patient was febrile and also had history of nausea and vomiting. On clinical examination, tenderness and guarding were observed at McBurney’s point.

**Figs 1A and B:**
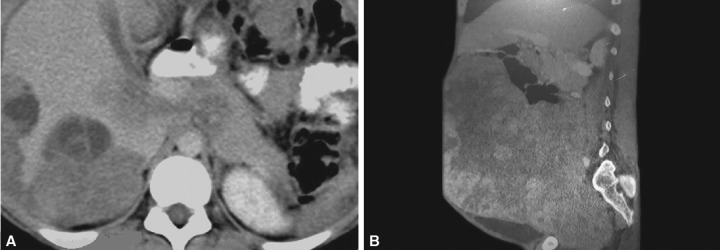
(A) Computed tomography (CT) scan (transverse view) showing scalloping of visceral surface due to compression by the peritoneal implants and (B) CT scan (lateral view) showing hyperdense mucinous ascites filling the peritoneal, appendix not visualized

Routine blood tests showed leukocytosis and raised erythrocyte sedimentation rate (ESR), while other hematological parameters were within normal limits. Chest X-ray were unremarkable while on plain X-rays abdomen (erect and supine), dilated intestinal loops were seen. Computed tomography (CT) scan revealed scalloping of visceral surface and hyperdense mucinous ascites filling the peritoneal cavity, bowel loops showing external compression by peritoneal implants, appendix was not visualized (Figs 1A and B).

Emergency laparotomy was done through a midline incision. Peroperative examination revealed, ruptured cystic mass (appendix) with adherent mucus material, attached to the dilated, tensed and inflamed cecum in the right iliac fossa. The small bowel appeared normal except for the mild dilatation of distal 8 cm of the terminal ileum. Pools of mucin were seen within the peritoneal fat and on the serosal surface of the viscera. No gross abnormality was visualized in the retroperitoneal organs, surgical debulking including appendectomy with partial resection of the attached cecum and extensive removal of gelatinous material was taken up. Subsequently, the peritoneal washing with normal saline was done, but chemotherapy was not given.

**Figs 2A and B:**
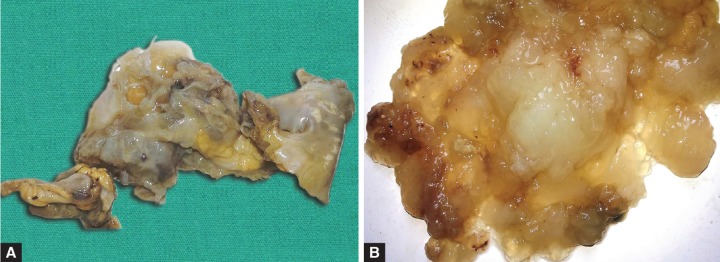
(A) Resected specimen showing a small tubular structure (appendix) of 2 × 1 cm attached to a ruptured distorted mass measuring 7 × 6 × 1 cm with abundant mucin adhered to the mucosal as well as serosal surface and (B) pool of acellular mucin

All the tissues removed along with the gelatinous material were submitted for histopathological examination. Gross examination revealed dilatation of the cecal area, measuring 10 × 9 × 9 cm with very scant amount of mucin in the lumen. The appendix was received as a small tubular structure of 2 × 1 cm attached to a ruptured distorted mass measuring 7 × 6 × 1 cm (Fig. 2A) with abundant mucin adhered to the mucosal as well as serosal surface. We also received large amount of mucin (Fig. 2B).

**Figs 3A to E:**
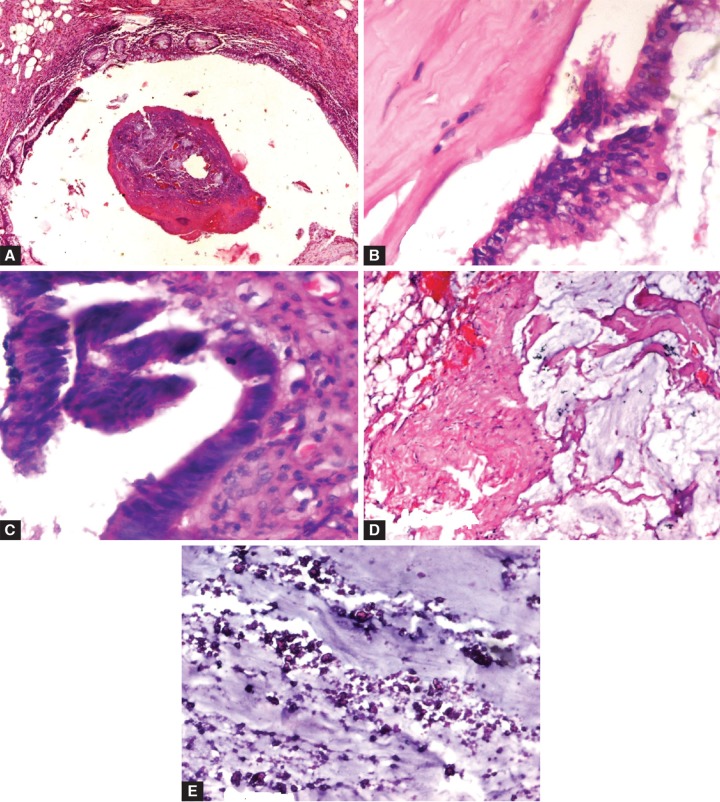
(A) Disturbed histomorphological characteristics of an appendix, with the loss of lymphoid follicles in the submucosa, (B) replacement of the normal appendiceal epithelium by mucin-producing columnar glandular epithelium, (C) part of the appendix showing villous adenoma-like dysplastic epithelium, the nuclei of the neoplastic cells were elongated, hyperchromatic and pseudostratified at places, (D) lakes of mucin seen dissecting the serosal fibrofatty tissue and (E) dystrophic calcification was in the acellular mucin pool

On microscopic examination, normal histomor-phological characteristics of an appendix were disturbed with the loss of lymphoid follicles in the submucosa (Fig. 3A) and replacement of the normal appendiceal epithelium by mucin-producing columnar glandular epithelium (Fig. 3B). Part of appendix showed villous adenoma like dysplastic epithelium, the nuclei of the neoplastic cells were elongated, hyperchromatic and pseudostratified at places (Fig. 3C). The muscularis propia was replaced by fibrotic, hyalinized tissue. Lakes of mucin seen dissecting the serosal fibrofatty tissue (Fig. 3D) and dystrophic calcification was in the acellular mucin pool (Fig. 3E). Cecal mucosa showed edema and transmural inflammatory infiltrates. Regional lymph nodes did not show any metastatic deposits. Correlating the clinical, CT, peroperative and histopathological findings, the diagnosis of LAMN neoplasm was reached.

The patient was discharged after a week stay in hospital in a satisfactory condition.

## DISCUSSION

Tumors of the appendix are rare clinical entities comprising of less than 2% of all appendectomies^5^ with an approximately 1% reported prevalence of LAMN.^1^ Classification of appendiceal mucinous neoplasms has been a difficult job because of several different reasons. Cyto-logically, LAMN usually behave as a benign neoplasm, however, as soon as the mucin or adenomatous epithelium escapes the appendiceal confines, a significant rise in morbidity and mortality is noticed even the cytology still remains of low-grade tumor. World Health Organization (WHO) classify appendiceal mucinous neoplasm^6^ in following groups:

- Adenocarcinoma
- Low-grade appendiceal mucinous neoplasm
- Mucinous adenocarcinoma
- Signet ring cell carcinoma
- Undifferentiated carcinoma

Pai et al (2009) evaluated 116 cases of appendiceal mucinous neoplasms and derived a succinct set of histologic features with important prognostic implication.^7^ Subsequently, they categorized mucinous appendiceal neoplasm into following four groups:

1. Mucinous (cyst) adenoma: Low-grade mucinous neoplasm confined to the appendix.
2. Low-grade mucinous neoplasms with low risk of recurrence (LG-LR): Features identical to mucinous adenoma, with the added component of acellular extra appendiceal mucin.
3. Low-grade mucinous neoplasms with high risk of recurrence (LG-HR): It is similar to LG-LR, but the extra appendiceal mucin contained neoplastic epithelium.
4. Mucinous adenocarcinoma: It shows presence of invasion; usually, but not always they exhibit high grade cytology and a complex architecture.

Approximately, 25% of appendiceal mucinous neoplasms are asymptomatic and found incidentally either on abdominal imaging or during surgery.^8^ Occasionally, these tumors may present with intestinal obstruction, intussusception, gastrointestinal bleeding and extrinsic ureteral compression. Rupture or perforation of the appendiceal wall, leads to dissemination of septic or neoplastic contents, resulting in localized or diffuse peritonitis or pseudomyxoma peritonei respectively. Pseudomyxoma peritonei is a pathologic entity having a variable clinical consequences; if this condition is caused by a high-grade appendiceal tumors, it is usually associated with a worse prognosis.^9^

Pseudomyxoma peritonei is distinctly defined entity characterized by the presence of intraperitoneal mucin, with or without associated mucin-producing epithelium. Not infrequently this condition is accompanied by fibrosis and granulation tissue formation.^2^ It is speculated that the mucin may spread to the peritoneal cavity causing pseudomyxoma peritonei, either by mucus tracking throughout the dilated, thinned appendiceal wall or through the appendiceal diverticulum. However the actual mechanism by which mucin traverses the app-endiceal wall in benign or low-grade neoplasm is still not fully illustrated, especially in cases in which definite invasion of the appendiceal wall is not observed.^10^ In our case, it occurred through ruptured thin walled appendix.

Due to frequent association of ovarian neoplasm causing pseudomyxoma peritonei, it should be kept in mind that, in all the cases of female with appendiceal mucinous neoplasms, the ovaries should be meticulously examined, similarly in all cases of ovarian mucinous neoplasms, the appendix should be looked for. Various surgical procedures can be implemented in managing mucinous appendiceal neoplasm complicated with pseudomyxoma peritonei, such as simple appendectomy, appendectomy with partial resection of the cecum, ileocecal resection and right hemicolectomy, performed either by open or laparoscopic approach. The histologic grade of peritoneal disease is considered as extremely important criteria. Patients with low-grade tumors may benefit from aggressive treatment that combines chemotherapy along with cytoreductive surgery, whereas high-grade tumor probably has a better response with systemic chemotherapy that includes peritoneal space washing with heated mitomycin solution or postoperative infusion of fluorouracil. Pseudomyxoma peritonei with scant, low-grade epithelium on microscopy has a good prognosis, while pseudomyxoma peritonei with abundant, high-grade (carcinomatous) epithelium has been found to have a bad prognostic outcome.

## CONCLUSION

- This case emphasizes the existence of a distinct subset of appendiceal mucinous tumor that lacks usual forms of destructive invasion, but has a propensity to spread to the peritoneal cavity, viscera and ovaries progressing to the clinical syndrome known as pseudo-myxoma peritonei.
- LAMN with pseudomyxoma peritonei can result in the demise of the patient, despite the bland histology of the mucinous epithelium.
- Grading of epithelium in pseudomyxoma/peritoneal mucinosis is important for management and prognosis.
- Clear communication between the pathologists and clinicians and their collaborative effort is therefore a prerequisite in order to reach to a confirmatory diagnosis.
